# Determinando Percentis do Risco Cardiovascular Aterosclerótico de acordo com Sexo e Idade numa População Saudável Brasileira

**DOI:** 10.36660/abc.20220552

**Published:** 2023-06-20

**Authors:** Fernando Yue Cesena, Nea Miwa Kashiwagi, Carlos Andre Minanni, Raul D. Santos

**Affiliations:** 1 Hospital Israelita Albert Einstein São Paulo SP Brasil Hospital Israelita Albert Einstein, São Paulo, SP – Brasil; 2 InCor Faculdade de Medicina Universidade de São Paulo São Paulo SP Brasil Instituto do Coração (InCor), Faculdade de Medicina da Universidade de São Paulo, São Paulo, SP – Brasil

**Keywords:** Fatores de Risco de Doenças Cardíacas, Medição de Risco, Doenças Cardiovasculares

## Abstract

**Fundamento:**

Expressar o risco de doença cardiovascular aterosclerótica (DCVA) em percentis da distribuição por sexo e idade pode proporcionar uma melhor percepção do risco.

**Objetivos:**

Determinar os percentis da distribuição do risco de DCVA em 10 anos segundo sexo e idade em uma amostra da população brasileira; caracterizar indivíduos com baixo risco em 10 anos, mas em alto percentil de risco.

**Métodos:**

Analisamos indivíduos de 40 a 75 anos que realizaram avaliações de saúde de rotina de 2010 a 2020. Foram excluídos indivíduos com DCVA clínica conhecida, diabetes mellitus, doença renal crônica ou LDL-colesterol ≥ 190 mg/dL. O risco de DCVA em 10 anos foi calculado pelas equações das coortes agrupadas do American College of Cardiology/American Heart Association. Foi utilizada a regressão polinomial local para determinar os percentis de risco. Valores de p bilateral < 0,050 foram considerados estatisticamente significativos.

**Resultados:**

Nossa amostra incluiu 54.145 atendimentos (72% do sexo masculino, idade mediana [intervalo interquartil] 48 [43; 53] anos). Construímos gráficos específicos por sexo traçando a idade contra o risco de DCVA correspondente aos percentis 10, 25, 50, 75 e 90. A maioria dos homens até 47 anos e mulheres até 59 anos acima do percentil 75 apresentaram risco em 10 anos < 5%. Indivíduos com baixo risco em 10 anos e percentil de risco ≥ 75 apresentaram alta prevalência de excesso de peso e níveis medianos (intervalos interquartis) de LDL-colesterol de 136 (109; 158) mg/dL (sexo masculino) e 126 (105; 147) mg/dL (sexo feminino).

**Conclusões:**

Estabelecemos percentis de risco de DCVA segundo sexo e idade em uma grande amostra da população brasileira. Essa abordagem pode aumentar a conscientização sobre o risco e ajudar a identificar pessoas mais jovens com baixo risco em 10 anos que podem se beneficiar de um controle mais agressivo dos fatores de risco.

## Introdução

A estratificação do risco cardiovascular é uma etapa fundamental para orientar estratégias de prevenção de eventos clínicos. O uso de escores de risco é amplamente recomendado pelas diretrizes de dislipidemia.^1-3^ Tanto a decisão de iniciar um medicamento hipolipemiante quanto as metas de colesterol da lipoproteína de baixa densidade (LDL-c) são estabelecidas a partir do risco absoluto de doença cardiovascular aterosclerótica (DCVA) em 10 anos. No entanto, a expressão do risco absoluto pode ser de difícil interpretação para os pacientes, comprometendo a conscientização do risco e a adesão ao tratamento. Além disso, a categorização de risco pode ser enganosa. Pessoas mais jovens com fatores de risco graves e não controlados podem ser rotuladas como de baixo risco devido à idade, mas podem ter um risco muito maior do que seus similares do mesmo sexo e idade, especialmente a longo prazo.

Uma proposta para melhorar a comunicação sobre o risco é informar como o risco do paciente se compara com o risco de outras pessoas semelhantes. Esse procedimento pode ser feito calculando os percentis da distribuição de risco de DCVA específicos para sexo e idade.^4^ No Brasil, até onde sabemos, os percentis de risco de DCVA por sexo e idade, de acordo com escores contemporâneos, não foram determinados. No presente estudo, procuramos determinar esses percentis usando as equações das coortes agrupadas do American College of Cardiology/American Heart Association (ACC/AHA)^5^ em uma grande amostra da população brasileira. Também caracterizamos indivíduos com percentis de risco mais altos para sexo e idade (considerados candidatos a medidas preventivas mais agressivas), mas com baixo risco calculado em 10 anos, que podem não ser identificados pelas diretrizes e não receber aconselhamento e tratamento adequados. Finalmente, desenvolvemos uma ferramenta de planilha para calcular facilmente o risco de DCVA em 10 anos e o percentil correspondente para sexo e idade.

## Métodos

### Desenho e população do estudo

O presente estudo é uma análise retrospectiva de indivíduos que realizaram avaliação de saúde de rotina no Hospital Israelita Albert Einstein (São Paulo-SP, Brasil). Tipicamente, nossos pacientes são saudáveis e vêm ao serviço uma vez por ano. O programa de avaliação de saúde inclui anamnese, exame físico por um clínico e coleta de sangue, entre vários procedimentos, conforme descrito anteriormente.^6^ Dados clínicos, demográficos, antropométricos e laboratoriais são coletados em um banco de dados.

Incluímos todos os atendimentos que aconteceram entre 1º de janeiro de 2010 e 31 de dezembro de 2020. Quando um indivíduo compareceu ao serviço mais de uma vez, foram incluídos todos os atendimentos. As palavras “casos” e “atendimentos” são usadas como sinônimos neste estudo.

Nossa população de interesse foi a de indivíduos sem condições de alto risco para os quais as diretrizes contemporâneas recomendam o uso de escores para estratificar o risco e orientar a terapia.^1-3^ Dessa forma, excluímos casos na presença de qualquer um dos seguintes fatores:

- DCVA clínica conhecida (por exemplo, infarto do miocárdio prévio, acidente vascular cerebral isquêmico de origem aterosclerótica ou procedimento de revascularização arterial);
- diabetes mellitus (diagnóstico autorreferido, glicemia de jejum ≥ 126 mg/dL ou hemoglobina glicada ≥ 6,5%);
- taxa de filtração glomerular estimada < 60 mL/min/1,73 m^2^ (de acordo com as equações para taxa de filtração glomerular da Chronic Kidney Disease Epidemiology Collaboration de 2021);^7^
- LDL-c (ou LDL-c estimado sem medicação) ≥ 190 mg/dL;
- idade < 40 anos ou > 75 anos (fora da faixa-alvo para o uso das equações das coortes agrupadas, conforme recomendado pela diretriz da AHA/ACC de 2018);^2^
- valores faltantes que não permitiram o cálculo do risco de DCVA.

Quando o participante referia uso de hipolipemiante (quase sempre uma estatina), estimamos o nível de LDL-c sem medicação multiplicando o LDL-c por um fator de conversão de 1,69, correspondendo a uma redução de 41% do LDL-c, que é a alteração média proporcionada por uma dose diária de 40 mg de sinvastatina.^8^ Essa dose de estatina foi escolhida com base na recomendação da Atualização da Diretriz Brasileira de Dislipidemias de usar um fator de conversão para estimar o nível de colesterol total sem medicação em usuários de estatina.^1^

O protocolo foi aprovado pelo Comitê de Ética em Pesquisa do Hospital Israelita Albert Einstein (São Paulo-SP, Brasil, CAAE 49545221.0.0000.0071). O Comitê de Ética aprovou a dispensa do consentimento informado por escrito com base na inviabilidade de obter o consentimento de milhares de participantes retrospectivamente. Além disso, o estudo é meramente observacional e a apresentação dos resultados não permite a identificação dos sujeitos.

### Estimativa do risco de DCVA em 10 anos

As equações das coortes agrupadas do ACC/AHA predizem o risco em 10 anos de eventos graves de DCVA (morte coronariana, infarto do miocárdio não fatal, acidente vascular cerebral fatal ou não fatal) a partir das seguintes variáveis: sexo, idade, raça, níveis de colesterol total e de colesterol da lipoproteína de alta densidade (HDL-c), pressão arterial sistólica, uso de medicamento anti-hipertensivo, presença de diabetes mellitus e presença de tabagismo.^5^ Utilizamos as equações para a raça branca, pois a grande maioria de nossos pacientes é branca e não estavam disponíveis informações sobre a raça de cada indivíduo. Quando o paciente estava tomando um hipolipemiante, o valor do colesterol total foi multiplicado por 1,43 para estimar o nível sem o medicamento, conforme procedimento recomendado pela Atualização da Diretriz Brasileira de Dislipidemias.^1^ Esse fator de conversão é derivado de dados de ensaios clínicos^9^ e corresponde aproximadamente a uma redução de 31% no nível de colesterol total proporcionada por uma dose diária de 40 mg de sinvastatina.^8^ Informações sobre a medicação específica e a dosagem utilizadas pelos participantes deste estudo não estavam disponíveis.

As categorias de risco foram definidas de acordo com a diretriz de colesterol da AHA/ACC de 2018 da maneira seguinte: risco baixo, limítrofe, intermediário e alto se o risco de DCVA em 10 anos foi < 5%, entre 5% e < 7,5%, entre 7,5% e < 20%, e ≥ 20%, respectivamente.^2^

### Apresentação de dados e análises estatísticas

As variáveis categóricas foram expressas como número de observações e porcentagens, enquanto as variáveis contínuas foram apresentadas como medianas e intervalos interquartis ou médias e desvios padrão. Os dados foram comparados usando o teste qui-quadrado de Pearson (variáveis categóricas), análise de variância de uma via (ANOVA, variáveis contínuas com distribuição normal) e o teste de Kruskal-Wallis (variáveis contínuas com distribuição não normal). O teste post-hoc de Games-Howell foi realizado após a ANOVA, pois não assume variâncias e tamanhos amostrais iguais. O método Dwass-Steel-Critchlow-Fligner foi usado em comparações pareadas após o teste de Kruskal-Wallis. Na comparação de amostras pareadas, utilizamos o teste de McNemar (variáveis categóricas), o teste t de amostras pareadas (variáveis contínuas com distribuição normal) e o teste de Wilcoxon (variáveis contínuas com distribuição não normal). A normalidade foi avaliada por inspeção visual de histogramas e gráficos quantil-quantil. Valores de p < 0,050 em testes bicaudais foram considerados estatisticamente significativos.

### Cálculo dos percentis da distribuição de risco de doença cardiovascular aterosclerótica

O método para determinar os percentis da distribuição do risco de DCVA segundo sexo e idade foi baseado nos procedimentos descritos para calcular os percentis da distribuição da calcificação da artéria coronária (CAC) no Multi-Ethnic Study of Atherosclerosis (MESA)^10^ e no Estudo Longitudinal de Saúde do Adulto (ELSA-Brasil).^11^

### As análises para cada sexo foram realizadas separadamente.

Primeiro, estimamos o risco de DCVA em 10 anos transformado em log para cada idade usando uma regressão polinomial local com suavização (LOESS, do inglês *locally estimated scatterplot smoothing*), com banda (span) de 0,7. A razão para transformar o risco em log reside no fato de que a distribuição do risco é muito assimétrica. Em seguida, calculamos os resíduos dos riscos log-transformados, significando a diferença entre cada observação e o valor estimado para a idade pelo modelo de regressão. Os resíduos nas vizinhanças de cada idade foram classificados e utilizados para a determinação dos percentis. O percentil *x* para uma determinada idade *y* foi o risco log-transformado estimado para essa idade adicionado ao percentil *x* dos resíduos na vizinhança de *y*, que foi arbitrariamente definido como um intervalo de 5 anos reunindo observações suficientes para permitir uma determinação precisa dos percentis. Por exemplo, para determinar os percentis de indivíduos de 50 anos, reunimos e classificamos os resíduos de pessoas de 48 a 52 anos. Finalmente, os percentis 10, 25, 50, 75 e 90 de cada idade foram retro-transformados para os riscos correspondentes de DCVA.

Em seguida, plotamos a idade contra o risco de DCVA correspondente aos percentis 10, 25, 50, 75 e 90. Linhas de tendência polinomiais de sexto grau para cada um dos percentis foram adicionadas ao gráfico e os valores correspondentes de R-quadrado foram determinados.

Definimos arbitrariamente alto percentil de risco como sendo percentil ≥ 75, significando um grupo que pode se beneficiar de um controle mais agressivo dos fatores de risco, independentemente do risco calculado de DCVA.

Por fim, construímos uma ferramenta que calcula o risco de DCVA em 10 anos pelas equações das coortes agrupadas e o percentil correspondente de acordo com sexo e idade com base na distribuição de risco em nossa população de estudo.

O software R versão 4.0.0 (R Foundation for Statistical Computing, Viena, Áustria) e Microsoft® Excel® para Microsoft 365 MSO (Versão 2202) foram usados para gerenciamento dos dados, determinação dos percentis, construção dos gráficos e desenvolvimento da calculadora.

## Resultados

**Figura 1:**
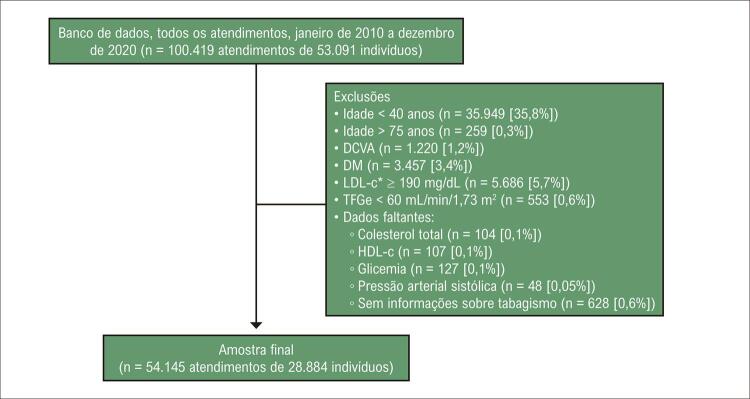
– Fluxograma dos casos incluídos e excluídos. As exclusões não são mutuamente exclusivas. * Em participantes que relataram uso de medicamento hipolipemiante, foi considerado um nível estimado de LDL-c sem medicação. DCVA: doença cardiovascular aterosclerótica; DM: diabetes mellitus; HDL-c: colesterol da lipoproteína de alta densidade; LDL-c: colesterol da lipoproteína de baixa densidade; TFGe: taxa de filtração glomerular estimada.

A Figura 1 apresenta o fluxograma dos casos incluídos e excluídos. A amostra final compreendeu 54.145 atendimentos de 28.884 participantes (média de 1,9 atendimentos por indivíduo, variando de 1 a 12 atendimentos).

Nossa população de estudo foi caracterizada por uma preponderância do sexo masculino (72%) e de indivíduos de meia-idade com baixo risco de DCVA (Tabela 1). As características basais de acordo com a categoria de risco de DCVA são apresentadas na Tabela Suplementar 1. O número de pacientes do sexo masculino e feminino na população do estudo, de acordo com a idade, está detalhado na Tabela Suplementar 2.

**Tabela 1:** – Características da população do estudo

Característica	Geral (n = 54.145)	Masculino (n = 39.091)	Feminino (n = 15.054)
Idade (anos)	48 (43, 53)	48 (43, 53)	47 (43, 52)
IMC (kg/m^2^)	26,4 (24,1; 29,0)	26,9 (24,9; 29,4)	24,5 (22,3; 27,5)
Sobrepeso/obesidade*	35.892 (66%)	29.033 (74%)	6.859 (46%)
Hipertensão arterial	14.199 (26%)	11.759 (30%)	2.440 (16%)
Pressão arterial sistólica (mmHg)	120 (110; 123)	120 (110; 125)	110 (102; 120)
Pressão arterial diastólica (mmHg)	80 (70; 80)	80 (70; 80)	70 (70; 80)
Medicamento hipolipemiante	7.410 (14%)	6.323 (16%)	1.087 (7,2%)
Colesterol total (mg/dL)	189 (167; 214)	189 (166; 214)	188 (167; 211)
LDL-c (mg/dL)	114 (94; 137)	117 (96; 139)	109 (90; 130)
HDL-c (mg/dL)	48 (40; 58)	45 (39; 53)	58 (49; 69)
Triglicérides (mg/dL)	108 (78; 153)	118 (86; 165)	86 (65; 118)
Glicemia (mg/dL)	86 (81; 92)	88 (83; 93)	83 (78; 88)
HbA1c (%)†	5,4 (5,2; 5,6)	5,4 (5,2; 5,7)	5,4 (5,2; 5,6)
Tabagismo atual	3.881 (7,2%)	2.955 (7,6%)	926 (6,2%)
Risco de DCVA em 10 anos (%)	2,3 (1,1; 4,8)	3,1 (1,7; 5,9)	0,7 (0,4; 1,5)
**Categoria de risco de DCVA**			
Baixo	41.506 (77%)	27.036 (69%)	14.470 (96%)
Limítrofe	5.693 (11%)	5.355 (14%)	338 (2,2%)
Intermediário	6.479 (12%)	6.251 (16%)	228 (1,5%)
Alto	467 (0,9%)	449 (1,1%)	18 (0,1%)

Dados expressos como mediana (intervalo interquartil) ou frequência (%). * IMC ≥ 25 kg/m^2^. † Com base em 45.533 (84,1%) casos com informações disponíveis. DCVA: doença cardiovascular aterosclerótica; HbA1c: hemoglobina glicada; HDL-c: colesterol da lipoproteína de alta densidade; IMC: índice de massa corporal; LDL-c: colesterol da lipoproteína de baixa densidade.

A Tabela Suplementar 3 mostra uma comparação entre os primeiros e últimos atendimentos entre 21.178 indivíduos com avaliações repetidas. A proporção de participantes usando medicação hipolipemiante ou anti-hipertensiva aumentou; o nível médio de LDL-c diminuiu e a pressão arterial média permaneceu dentro dos limites normais.

**Figura 2:**
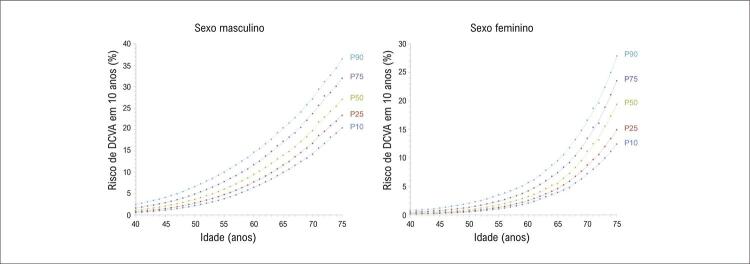
– Curvas de percentis do risco de DCVA em 10 anos, segundo sexo e idade. P10, P25, P50, P75 e P90 representam os percentis 10, 25, 50, 75 e 90, respectivamente. Linhas de tendência polinomiais de sexto grau são exibidas. Todos os R-quadrado são ≥ 0,9992. DCVA: doença cardiovascular aterosclerótica.

A distribuição do risco de DCVA em 10 anos de acordo com a idade é mostrada na Figura Suplementar 1 (sexo masculino) e na Figura Suplementar 2 (sexo feminino). Os valores correspondentes aos percentis 10, 25, 50, 75 e 90 da distribuição do risco de DCVA em 10 anos, segundo sexo e idade, dados pela regressão LOESS, são apresentados na Tabela Suplementar 4. A Figura 2 graficamente representa esses pontos com as resultantes curvas de percentis.

**Figura 3:**
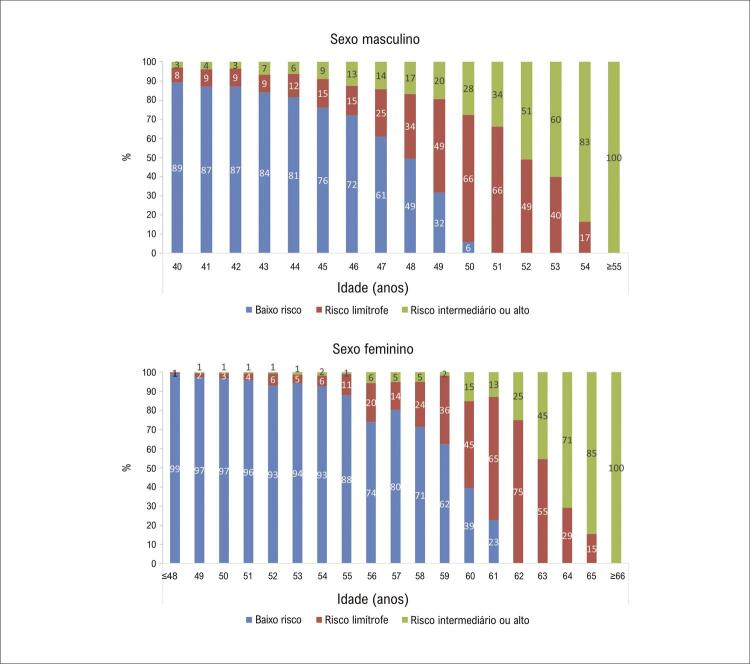
– Distribuição das categorias de risco de doença cardiovascular aterosclerótica em 10 anos segundo sexo e idade entre indivíduos no percentil de risco ≥ 75.

Entre os casos no percentil de risco ≥ 75, a maioria dos homens até a idade de 47 anos e quase metade daqueles na idade de 48 anos estavam na categoria de baixo risco de DCVA em 10 anos (Figura 3). Entre as mulheres no percentil ≥ 75, a maioria até a idade de 59 anos e quase 40% daquelas com 60 anos eram de baixo risco (Figura 3).

As principais características dos indivíduos com baixo risco de DCVA em 10 anos e percentil de risco ≥ 75 estão detalhadas na Tabela 2, Tabelas Suplementares 5 e 7 (de acordo com o tabagismo) e Tabelas Suplementares 6 e 8 (de acordo com a ausência ou presença de hipertensão arterial). Esse subgrupo de indivíduos de meia-idade foi caracterizado por maior prevalência de excesso de peso, hipertensão arterial e tabagismo em comparação com toda a população do estudo. O nível mediano de HDL-c estava abaixo do ideal em ambos os sexos e o nível mediano dos triglicérides estava elevado no sexo masculino. A mediana (intervalo interquartil) dos valores de LDL-c foi de 114 (98; 132) mg/dL e 113 (93; 130) mg/dL em homens e mulheres fumantes, respectivamente, e 133 (107; 154) mg/dL e 122 (101; 142) mg/dL em homens e mulheres com hipertensão arterial, respectivamente.

**Tabela 2:** – Características dos indivíduos com baixo risco de DCVA em 10 anos e percentil de risco ≥ 75, segundo sexo

Característica	Masculino (n = 4.264)	Feminino (n = 3.625)
Idade (anos)	43 (41; 46)	46 (42; 50)
IMC (kg/m^2^)	28,2 (26,1; 30,7)	26,4 (23,8; 29,6)
Sobrepeso/obesidade*	3.643 (85%)	2.349 (65%)
Hipertensão arterial	1.690 (40%)	1.164 (32%)
Pressão arterial sistólica (mmHg)	120 (118; 130)	120 (110; 125)
Pressão arterial diastólica (mmHg)	80 (80; 86)	80 (70; 80)
Colesterol total (mg/dL)	212 (185; 235)	200 (177; 223)
LDL-c (mg/dL)	136 (109; 158)	126 (105; 147)
HDL-c (mg/dL)	39 (34; 44)	48 (41; 56)
Triglicérides (mg/dL)	171 (126; 235)	114 (84; 156)
Glicemia (mg/dL)	88 (83; 94)	84 (79; 90)
HbA1c (%)	5,4 (5,2; 5,7)	5,4 (5,2; 5,7)
Tabagismo atual	642 (15%)	721 (20%)
Risco de DCVA em 10 anos (%)	3,26 (2,49; 4,04)	1,30 (0,83; 2,25)

Dados expressos como mediana (intervalo interquartil) ou frequência (%). * IMC ≥ 25 kg/m^2^. DCVA: doença cardiovascular aterosclerótica; HbA1c: hemoglobina glicada; HDL-c: colesterol da lipoproteína de alta densidade; IMC: índice de massa corporal; LDL-c: colesterol da lipoproteína de baixa densidade.

A calculadora do risco de DCVA em 10 anos está disponível para download como um arquivo Microsoft^®^ Excel^®^ suplementar. A ferramenta automaticamente calcula e exibe graficamente o percentil de risco de DCVA para o sexo e idade correspondentes (Figura Central). O usuário também pode estimar o nível de colesterol total quando o paciente estiver tomando medicamentos hipolipemiantes.

**Figura Central:**
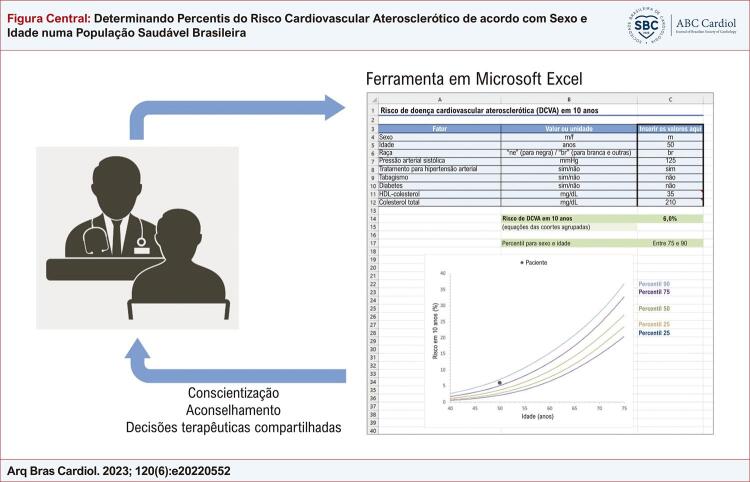
: Determinando Percentis do Risco Cardiovascular Aterosclerótico de acordo com Sexo e Idade numa População Saudável Brasileira

Aplicação clínica da ferramenta em Microsoft Excel para calcular o risco de doença cardiovascular aterosclerótica em 10 anos e o percentil para sexo e idade. No exemplo descrito, o risco em 10 anos não é alto, mas o percentil para sexo e idade é maior que 75. Também é possível prever o risco futuro em 10 anos se o paciente persistir no mesmo percentil. Os percentis podem facilitar a comunicação de risco, aumentar a conscientização do paciente, ajudar o médico a aconselhar o paciente e contribuir para decisões terapêuticas compartilhadas.

## Discussão

Estabelecemos percentis da distribuição de risco de DCVA em 10 anos específicos para sexo e idade, de acordo com as equações das coortes agrupadas do ACC/AHA, em uma grande amostra da população brasileira. Verificamos que a maioria dos homens até 47 anos e mulheres até 59 anos acima do percentil 75 de risco tinham um risco em 10 anos < 5% (categoria de baixo risco), um limiar frequentemente usado para adiar a terapia com estatina na prevenção primária com LDL-c abaixo de 190 mg/dL.^2^ Também fornecemos uma calculadora fácil de usar para o risco de DCVA e o percentil correspondente para sexo e idade, o que pode facilitar o uso clínico desta abordagem (Figura Central).

O presente estudo pode ser inserido em um contexto mais amplo de como eficientemente comunicar o risco cardiovascular estimado, o que é fundamental para o engajamento do paciente e a implementação bem-sucedida de ações preventivas, especialmente no cenário de decisões compartilhadas. Muitas pessoas podem ver o risco absoluto como um número abstrato e sem sentido. Além disso, a percepção errônea do risco é comum, inclusive entre indivíduos que frequentaram nosso serviço.^12^ Do ponto de vista do paciente, saber como seu risco se compara a seus pares pode aumentar a conscientização do risco quando o percentil for alto.

Outra proposta para facilitar a compreensão do risco cardiovascular é calcular a "idade cardíaca" ou "idade vascular" como sinônimo da idade de alguém do mesmo sexo com o mesmo risco previsto, mas todos os fatores de risco em faixas normais.^13,14^ De fato, a expressão do risco como a idade cardíaca tem se mostrado eficaz no controle dos fatores de risco em comparação com a informação do risco absoluto ou orientação médica convencional.^15^ A idade vascular, entretanto, pode ser criticada por ser um cálculo grosseiro, visto que se baseia em valores normais escolhidos arbitrariamente. Além disso, o conceito de idade cardíaca não envolve uma comparação direta com os pares. Portanto, a idade vascular e os percentis de risco são intrinsecamente diferentes e ambos se complementam na tarefa de otimizar a comunicação de risco.

No contexto da prevenção cardiovascular, os percentis de risco compartilham semelhanças com os percentis de CAC específicos para idade, sexo e raça/etnia, que podem orientar as decisões clínicas.^16^ Da mesma forma, o conhecimento do percentil de risco pode refinar o atendimento ao paciente, auxiliando os profissionais de saúde na decisão sobre a intensidade das estratégias preventivas, a frequência da avaliação médica/laboratorial ou a necessidade de avaliação de risco complementar, por exemplo, com investigação de aterosclerose subclínica.

Os percentis de risco podem ser particularmente úteis em jovens nos quais o risco calculado em 10 anos pode ser baixo mesmo na presença de fatores de risco, porque a idade é o principal determinante do risco. Nossas análises identificam homens na faixa dos 40 anos e mulheres até aproximadamente 60 anos como faixas etárias nas quais um alto percentil de risco é mais frequentemente associado a um baixo risco em 10 anos. Propostas para orientar medidas preventivas, como a terapia com estatina, em indivíduos com baixo risco em 10 anos incluem a estimativa do risco de longo prazo e a redução do limiar de risco de 10 anos para classificar jovens como de alto risco.^14,17^ O uso de percentis de risco com base na distribuição de risco em 10 anos, como forma de espelhar indiretamente o risco ao longo da vida, pode ser visto como outra possibilidade atraente. Além disso, mostramos que os indivíduos de baixo risco/alto percentil de risco frequentemente têm excesso de peso e outras anormalidades metabólicas, o que é consistente com a evidência que associa a síndrome metabólica ao risco de DCVA.^18^ Por outro lado, estes indivíduos geralmente não têm LDL-c muito elevado, especialmente na presença de tabagismo ou hipertensão arterial. Portanto, uma estratégia de iniciação de estatina baseada apenas em níveis muito altos de LDL-c ou limiares de risco em 10 anos excluiria muitas pessoas com alto risco a longo prazo. Essa questão torna-se mais relevante no contexto de chamadas para usar preferencialmente a predição do risco ao longo da vida em vez do risco em 10 anos e tratar o risco de DCVA de forma mais intensa e precoce na vida.^17,19^

Com base nas equações das coortes agrupadas, Navar et al. estabeleceram percentis de risco de DCVA para sexo, idade e raça na população dos Estados Unidos.^4^ Podem ser observadas algumas diferenças com nossos resultados. Por exemplo, Navar et al. relataram os seguintes percentis 25, 50 e 75 para homens não negros de 55 anos: 4,9%, 7,0% e 10,2%, respectivamente.^4^ Em nossa população, os respectivos percentis foram 4,7%, 6,0% e 7,8%. Na população dos Estados Unidos, os percentis 25, 50 e 75 para mulheres não negras de 65 anos foram 5,1%, 6,9% e 9,9%, respectivamente, enquanto os respectivos números foram 4,6%, 5,4% e 7,4% em nossa população. Embora ambos estudos tenham utilizado abordagens metodológicas diferentes para determinar os percentis, essas diferenças podem refletir uma distribuição de risco verdadeiramente diversa nas populações, reforçando as limitações de extrapolar os resultados de um país para outro.

Uma questão relevante em nossa análise se refere à forma de lidar com participantes em uso de medicamento hipolipemiante. Ao contrário da medicação anti-hipertensiva, o uso de medicamentos hipolipemiantes não é uma variável nas equações das coortes agrupadas, pois essa terapia era relativamente incomum nas coortes de derivação.^5^ A simples exclusão desses indivíduos de nossas análises agiria contra o objetivo do estudo, que era determinar os percentis de risco em toda a população-alvo. Imputar o valor de colesterol total com medicação na equação de risco pode ser enganoso, subestimando o risco verdadeiro. Assim, optamos por usar um nível estimado de colesterol total sem medicação no cálculo de risco, conforme recomendado pela mais recente diretriz brasileira sobre dislipidemia.^1^ Acreditamos que essa abordagem foi a mais adequada para estabelecer percentis de risco populacional que podem ajudar os médicos clínicos a decidir se a terapia com estatina deve ser iniciada, um dos principais usos práticos da estratificação de risco de DCVA.

Um ponto forte do nosso estudo é que ele avalia uma grande amostra contemporânea, o que nos permitiu conduzir análises específicas por sexo e idade. No entanto, várias limitações devem ser destacadas. Primeiro, as equações das coortes agrupadas não foram validadas nem calibradas na população brasileira. Em particular, os efeitos independentes de raça, um componente-chave das equações das coortes agrupadas, em eventos cardiovasculares no Brasil são amplamente desconhecidos. Lotufo e Benseñor relataram maiores taxas de mortalidade por doença cardiovascular total e acidente vascular cerebral ajustadas por idade em pessoas negras;^20,21^ no entanto, essas análises não foram ajustadas para outros fatores de risco, como hipertensão arterial, colesterol total e tabagismo. Em segundo lugar, nossa amostra, composta em sua maioria por indivíduos brancos de alto nível socioeconômico que frequentaram um serviço privado em uma grande cidade da Região Sudeste, está longe de ser representativa da população brasileira. Vários estudos relatam uma menor carga de anormalidades lipídicas e de pressão arterial elevada em subgrupos com níveis socioeconômicos ou educacionais mais elevados no Brasil,^22-27^ o que pode ser, pelo menos em parte, explicado pelo uso mais frequente de medicamentos preventivos.^28^ Em nosso estudo, esse efeito do tratamento é provavelmente atenuado, pois a medicação anti-hipertensiva já é uma variável nas equações das coortes agrupadas, e aplicamos um fator de conversão para estimar o nível de colesterol total sem medicação. As taxas de tabagismo no Brasil também diminuem com o aumento de anos de escolaridade.^26,27^ Reconhecemos ainda que existem diferenças regionais na distribuição dos fatores de risco na população.^22,26,27^ Todos esses fatores potencialmente modificam os percentis de risco; portanto, é necessário praticar cautela ao aplicar os resultados deste estudo em outros cenários. Finalmente, a estimativa do risco cardiovascular por escores é apenas o primeiro passo na estratificação do risco. Os médicos clínicos devem ter em mente que vários fatores não contemplados nas equações de risco, como obesidade, história familiar de DCVA prematura, marcadores inflamatórios e CAC podem ser usados para identificar pessoas sob risco.

## Conclusões

Estabelecemos percentis da distribuição do risco de DCVA em 10 anos para sexo e idade em uma amostra grande, embora não representativa da população brasileira. A maioria dos homens até 47 anos de idade e mulheres até 59 anos acima do percentil 75 são categorizados como indivíduos de baixo risco pela Diretriz sobre o Manejo do Colesterol Sanguíneo da AHA/ACC de 2018. As pessoas de baixo risco/alto percentil de risco frequentemente apresentam anormalidades metabólicas e níveis de LDL-c não muito elevados. Esses indivíduos provavelmente têm alto risco de DCVA a longo prazo e podem ser candidatos a um controle mais agressivo dos fatores de risco, incluindo o início precoce da terapia com estatinas. A expressão de percentis de risco pode melhorar a comunicação do risco, aumentar a conscientização dos pacientes e a adesão a estratégias preventivas, e facilitar processos de tomada de decisão compartilhada.
