# Supplementary material for: Determinando Percentis do Risco Cardiovascular Aterosclerótico de acordo com Sexo e Idade numa População Saudável Brasileira
Source: Arq Bras Cardiol. 2023 Jun 20;120(6):e20220552. [Article in Portuguese] doi: 10.36660/abc.20220552 (PMC10344368; doi:10.36660/abc.20220552)
Supplement: Translation

# Translation: Determining Percentiles of Atherosclerotic Cardiovascular Risk According to Sex and Age in a Healthy Brazilian Population

**Authors:** Fernando Yue Cesena, Nea Miwa Kashiwagi, Carlos Andre Minanni, Raul D Santos

**Affiliations:** 1 Brazil Hospital Israelita Albert Einstein, São Paulo, SP – Brazil; 2 Brazil Instituto do Coração (InCor), Faculdade de Medicina da Universidade de São Paulo, São Paulo, SP – Brazil

**Keywords:** Heart Disease Risk Factors, Risk Assessment, Cardiovascular Diseases

## Abstract

**Background:**

Expressing the risk of atherosclerotic cardiovascular disease (ASCVD) as percentiles of the distribution according to sex and age may provide a better perception of the risk.

**Objectives:**

To determine percentiles of the 10-year ASCVD risk distribution according to sex and age in a sample of the Brazilian population; to characterize individuals at low 10-year risk but high risk percentile.

**Methods:**

We analyzed individuals aged 40 to 75 years who underwent routine health evaluations from 2010 to 2020. Persons with known clinical ASCVD, diabetes mellitus, chronic kidney disease, or LDL-cholesterol ≥ 190 mg/dL were excluded. The 10-year ASCVD risk was calculated by the ACC/AHA pooled cohort equations. Local polynomial regression was used to determine risk percentiles. Two-sided p-values < 0.050 were considered statistically significant.

**Results:**

Our sample comprised 54,145 visits (72% male, median age [interquartile range] 48 [43, 53] years). We constructed sex-specific graphs plotting age against ASCVD risk corresponding to the 10^th^, 25^th^, 50^th^, 75^th^, and 90^th^ percentiles. Most males up to 47 years and females up to 59 years above the 75^th^ percentile had a 10-year risk < 5%. Individuals at low 10-year risk and risk percentile ≥ 75^th^ had a high prevalence of excess weight and median (interquartile range) LDL-cholesterol levels 136 (109, 158) mg/dL (males) and 126 (105, 147) mg/dL (females).

**Conclusions:**

We established ASCVD risk percentiles according to sex and age in a large sample of the Brazilian population. This approach may increase risk awareness and help identify younger persons at low 10-year risk who may benefit from more aggressive risk factor control.

## Introduction

Cardiovascular risk stratification is a fundamental step to guide strategies to prevent clinical events. The use of risk scores is widely recommended by dyslipidemia guidelines.^1-3^ Both the decision to initiate a lipid-lowering drug and low-density lipoprotein cholesterol (LDL-c) targets are established from the absolute 10-year risk of atherosclerotic cardiovascular disease (ASCVD). However, the expression of the absolute risk may be difficult for patients to interpret, compromising the awareness of risk and adherence to treatment. Also, risk categorization may be misleading. Younger persons with severe, uncontrolled risk factors may be labeled as low-risk because of age, but may have a much higher risk than their counterparts of the same sex and age, especially in the long term.

A proposal to improve risk communication is informing how the patient´s risk compares with the risk of other similar persons. This procedure can be done by calculating sex- and age-specific percentiles of ASCVD risk distribution.^4^ In Brazil, to the best of our knowledge, percentiles of ASCVD risk for sex and age, according to contemporaneous scores, have not been determined. In this study, we sought to establish these percentiles, using the American College of Cardiology/American Heart Association (ACC/AHA) pooled cohort equations,^5^ in a large sample of the Brazilian population. We also characterized individuals at higher risk percentiles for sex and age (deemed candidates for more aggressive preventive measures), but at low calculated 10-year risk, who may be missed by guidelines and not adequately advised and treated. Finally, we developed a spreadsheet tool to easily calculate the 10-year ASCVD risk and the corresponding percentile for sex and age.

## Methods

### Design and study population

This study is a retrospective analysis of individuals who underwent a routine health evaluation at Hospital Israelita Albert Einstein (São Paulo, SP, Brazil). Typically, our patients are healthy and come to the service once a year. The health evaluation program includes anamnesis, physical examination by a clinician, and blood collection, among several procedures, as previously described.^6^ Clinical, demographic, anthropometric, and laboratory data are collected in a database.

We included all the visits that happened between January 1, 2010, and December 31, 2020. When one individual visited the service more than once, all the visits were included. “Cases” and “visits” are used as synonyms in this study.

Our population of interest was individuals without high-risk conditions for whom the contemporary guidelines recommend the use of scores to risk stratify and guide the therapy.^1-3^ Accordingly, we excluded cases in the presence of any of the following factors:

- known clinical ASCVD (e.g., previous myocardial infarction, ischemic stroke of atherosclerotic origin, or arterial revascularization procedure);
- diabetes mellitus (self-reported diagnosis, fasting glycemia ≥ 126 mg/dL, or glycated hemoglobin ≥ 6.5%);
- estimated glomerular filtration rate < 60 mL/min/1.73 m^2^ (according to the 2021 Chronic Kidney Disease Epidemiology Collaboration Equations for Glomerular Filtration Rate);^7^
- LDL-c (or estimated LDL-c without medication) ≥ 190 mg/dL;
- age < 40 years or > 75 years (out of the target range for the use of the pooled cohort equations as recommended by the 2018 AHA/ACC guideline);^2^
- missing values not allowing calculation of ASCVD risk.

When the participant reported use of a lipid-lowering drug (almost always a statin), we estimated the LDL-c level without medication by multiplying the LDL-c by a conversion factor of 1.69, corresponding to a 41% LDL-c reduction, which is the mean change provided by a daily dose of simvastatin 40 mg.^8^ This statin dose was chosen on the basis of a recommendation by the Update of the Brazilian Guideline on Dyslipidemia to use a conversion factor to estimate the total cholesterol level without medication in statin users.^1^

The protocol was approved by the Research Ethics Committee of the Hospital Israelita Albert Einstein (São Paulo, SP, Brazil, CAAE 49545221.0.0000.0071). The Ethics Committee approved a waiver of the written informed consent based on the unfeasibility of obtaining the consent of thousands of participants retrospectively. Moreover, the study is merely observational, and the presentation of results does not allow the identification of subjects.

### Estimation of the 10-year ASCVD risk

The ACC/AHA pooled cohort equations predict the 10-year risk of hard ASCVD events (coronary death, nonfatal myocardial infarction, fatal or nonfatal stroke) from the following variables: sex, age, race, total cholesterol and high-density lipoprotein cholesterol (HDL-c) levels, systolic blood pressure, use of antihypertensive medication, presence of diabetes mellitus, and presence of smoking.^5^ We used the equations for the White race since the great majority of our patients are White, and the information on each individual’s race was not available. When the individual was taking a lipid-lowering drug, the total cholesterol value was multiplied by 1.43 to estimate a level without the medication, according to the procedure recommended by the Update of the Brazilian Guideline on Dyslipidemia.^1^ This conversion factor derives from data from clinical trials^9^ and approximately corresponds to a 31% reduction in total cholesterol level proportioned by a daily dose of simvastatin 40 mg.^8^ Information on specific medication and dosage used by participants in this study was not available.

Risk categories were defined according to the 2018 AHA/ACC cholesterol guideline as follows: low, borderline, intermediate, and high risk if the 10-year ASCVD risk was < 5%, between 5% and < 7.5%, between 7.5% and < 20%, and ≥ 20%, respectively.^2^

### Data presentation and statistical analyses

Categorical variables were expressed as the number of observations and percentages, whereas continuous variables were shown as medians and interquartile ranges or means and standard deviations. Data were compared by the Pearson’s chi-squared test (categorical variables), the one-way analysis of variance (ANOVA, continuous variables with normal distribution), and the Kruskal-Wallis test by ranks (continuous variables with non-normal distribution). The Games-Howell post-hoc test was performed after ANOVA as it does not assume equal variances and sample sizes. The Dwass-Steel-Critchlow-Fligner method was used in pairwise comparisons after the Kruskal-Wallis test. When comparing paired samples, we used the McNemar test (categorical variables), the paired samples *t*-test (continuous variables with normal distribution), and the Wilcoxon signed-rank test (continuous variables with non-normal distribution). Normality was assessed by visual inspection of histograms and quantile-quantile plots. A p-value <0.050 in two-sided tests was considered statistically significant.

### Calculation of the percentiles of the atherosclerotic cardiovascular disease risk distribution

The method to determine the percentiles of the ASCVD risk distribution according to sex and age was based on the procedures described to calculate percentiles of the distribution of coronary artery calcification (CAC) in the Multi-Ethnic Study of Atherosclerosis (MESA)^10^ and the Brazilian Longitudinal Study of Adult Health (ELSA-Brasil).^11^

### Analyses for each sex were done separately.

We first estimated the log-transformed 10-year ASCVD risk for each age using locally estimated scatterplot smoothing (LOESS), that is, a local polynomial regression, with a span of 0.7. The reason to log-transform the risk resides in the fact that the risk distribution is very skewed. We then calculated the residuals of the log-transformed risks, meaning the difference between each observation and the estimated value for the age by the regression model. The residuals in the vicinity of each age were ranked and used for the determination of the percentiles. The percentile *x* for a given age *y* was the estimated log-transformed risk for that age added to the percentile *x* of the residuals in the vicinity of *y*, which was arbitrarily set as a 5-year range gathering enough observations to allow an accurate determination of the percentiles. For instance, to determine the percentiles for 50-year-old individuals, we put together and ranked the residuals of persons aged 48 to 52 years. Finally, each age's 10^th^, 25^th^, 50^th^, 75^th^, and 90^th^ percentiles were back-transformed to the corresponding ASCVD risks.

Next, we plotted age against the ASCVD risk corresponding to the 10^th^, 25^th^, 50^th^, 75^th^, and 90^th^ percentiles. Sixth-degree polynomial trendlines for each of the percentiles were added to the graph and corresponding R-squared values were determined.

We arbitrarily defined high risk percentile as ≥ 75^th^, meaning a group that may benefit from more aggressive risk factor control, independently of the calculated ASCVD risk.

Finally, we constructed a tool that calculates the 10-year ASCVD risk by the pooled cohort equations and the corresponding percentile according to sex and age based on the risk distribution in our study population.

The R software version 4.0.0 (R Foundation for Statistical Computing, Vienna, Austria) and Microsoft® Excel® for Microsoft 365 MSO (Version 2202) were used for data management, determination of the percentiles, graph construction, and the development of the calculator.

## Results

Figure 1 shows the flowchart of included and excluded cases. The final sample comprised 54,145 visits of 28,884 participants (average of 1.9 visits per individual, ranging from 1 to 12 visits).

**Figure 1:** – Flowchart of included and excluded cases. Exclusions are not mutually exclusive. * In participants reporting use of lipid-lowering medication, an estimated LDL-c level without medication was considered. ASCVD: atherosclerotic cardiovascular disease; DM: diabetes mellitus; eGFR: estimated glomerular filtration rate; HDL-c: high-density lipoprotein cholesterol; LDL-c: low-density lipoprotein cholesterol.

Our study population was characterized by a preponderance of male sex (72%), middle-aged individuals at low ASCVD risk (Table 1). Baseline characteristics according to the ASCVD risk category are shown in Supplemental Table 1. The number of males and females in the study population, according to age, is detailed in Supplemental Table 2.

**Table 1:** – Characteristics of the study population

Characteristic	Overall (n = 54,145)	Males (n = 39,091)	Females (n = 15,054)
Age (years)	48 (43, 53)	48 (43, 53)	47 (43, 52)
BMI (kg/m^2^)	26.4 (24.1, 29.0)	26.9 (24.9, 29.4)	24.5 (22.3, 27.5)
Overweight/obesity*	35,892 (66%)	29,033 (74%)	6,859 (46%)
Arterial hypertension	14,199 (26%)	11,759 (30%)	2,440 (16%)
Systolic blood pressure (mmHg)	120 (110, 123)	120 (110, 125)	110 (102, 120)
Diastolic blood pressure (mmHg)	80 (70, 80)	80 (70, 80)	70 (70, 80)
Lipid-lowering medication	7,410 (14%)	6,323 (16%)	1,087 (7.2%)
Total cholesterol (mg/dL)	189 (167, 214)	189 (166, 214)	188 (167, 211)
LDL-c (mg/dL)	114 (94, 137)	117 (96, 139)	109 (90, 130)
HDL-c (mg/dL)	48 (40, 58)	45 (39, 53)	58 (49, 69)
Triglycerides (mg/dL)	108 (78, 153)	118 (86, 165)	86 (65, 118)
Glycemia (mg/dL)	86 (81, 92)	88 (83, 93)	83 (78, 88)
HbA1c (%)†	5.4 (5.2, 5.6)	5.4 (5.2, 5.7)	5.4 (5.2, 5.6)
Current smoking	3,881 (7.2%)	2,955 (7.6%)	926 (6.2%)
10-year ASCVD risk (%)	2.3 (1.1, 4.8)	3.1 (1.7, 5.9)	0.7 (0.4, 1.5)
**ASCVD risk category**			
Low	41,506 (77%)	27,036 (69%)	14,470 (96%)
Borderline	5,693 (11%)	5,355 (14%)	338(2.2%)
Intermediate	6,479 (12%)	6,251 (16%)	228(1.5%)
High	467 (0.9%)	449 (1.1%)	18 (0.1%)

Data expressed as median (interquartile range) or frequency (%). * BMI ≥ 25 kg/m^2^. † Based on 45,533 (84.1%) cases with available information. ASCVD: atherosclerotic cardiovascular disease; BMI: body mass index; HbA1c: glycated hemoglobin; HDL-c: high-density lipoprotein cholesterol; LDL-c: low-density lipoprotein cholesterol.

**Table 2:** – Characteristics of individuals at low 10-year ASCVD risk and risk percentile ≥ 75**th**, according to sex

Characteristic	Males (n = 4,264)	Females (n = 3,625)
Age (years)	43 (41, 46)	46 (42, 50)
BMI (kg/m^2^)	28.2 (26.1, 30.7)	26.4 (23.8, 29.6)
Overweight/obesity*	3,643 (85%)	2,349 (65%)
Arterial hypertension	1,690 (40%)	1,164 (32%)
Systolic blood pressure (mmHg)	120 (118, 130)	120 (110, 125)
Diastolic blood pressure (mmHg)	80 (80, 86)	80 (70, 80)
Total cholesterol (mg/dL)	212 (185, 235)	200 (177, 223)
LDL-c (mg/dL)	136 (109, 158)	126 (105, 147)
HDL-c (mg/dL)	39 (34, 44)	48 (41, 56)
Triglycerides (mg/dL)	171 (126, 235)	114 (84, 156)
Glycemia (mg/dL)	88 (83, 94)	84 (79, 90)
HbA1c (%)	5.4 (5.2, 5.7)	5.4 (5.2, 5.7)
Current smoking	642 (15%)	721 (20%)
10-year ASCVD risk (%)	3.26 (2.49, 4.04)	1.30 (0.83, 2.25)

Data expressed as median (interquartile range) or frequency (%). * BMI ≥ 25 kg/m^2^. ASCVD: atherosclerotic cardiovascular disease; BMI: body mass index; HbA1c: glycated hemoglobin; HDL-c: high-density lipoprotein cholesterol; LDL-c: low-density lipoprotein cholesterol.

Supplemental Table 3 shows a comparison between the first and the last visits among 21,178 individuals with repeated evaluations. The proportion of participants on lipid-lowering or antihypertensive medication increased; the mean LDL-c level decreased, and the average blood pressure remained within normal ranges.

The distribution of the 10-year ASCVD risk according to age is depicted in Supplemental Figure 1 (males) and Supplemental Figure 2 (females). Values corresponding to the 10^th^, 25^th^, 50^th^, 75^th^, and 90^th^ percentiles of the distribution of the 10-year ASCVD risk, according to sex and age, given by the LOESS regression, are shown in the Supplemental Table 4. Figure 2 graphically represents these points with the resultant percentile curves.

**Figure 2:** – Curves of the 10-year ASCVD risk percentiles, according to sex and age. P10, P25, P50, P75, and P90 represent the 10th, 25th, 50th, 75th, and 90th percentiles, respectively. Sixth-degree polynomial trendlines are shown. All R-squared are ≥ 0.9992. ASCVD: atherosclerotic cardiovascular disease.

Among cases in the risk percentile ≥ 75^th^, most males up to the age of 47 years and almost half of those at the age of 48 years were in the category of low 10-year ASCVD risk (Figure 3). Among females in the percentile ≥ 75^th^, most up to the age of 59 years and almost 40% of those at the age of 60 years were low-risk individuals (Figure 3).

**Figure 3:** – Distribution of categories of the 10-year risk of atherosclerotic cardiovascular disease according to sex and age among individuals in the risk percentile ≥ 75th.

The main characteristics of individuals at low 10-year ASCVD risk and risk percentile ≥ 75^th^ are detailed in Table 2, Supplemental Tables 5 and 7 (according to smoking status), and Supplemental Tables 6 and 8 (according to the absence or presence of arterial hypertension). This subgroup of middle-aged individuals was characterized by a higher prevalence of excess weight, arterial hypertension, and smoking compared to the whole study population. Median HDL-c was below ideal values in both sexes and the median triglyceride level was elevated in males. Median (interquartile range) LDL-c values were 114 (98, 132) mg/dL and 113 (93, 130) mg/dL in smoker males and females, respectively, and 133 (107, 154) mg/dL and 122 (101, 142) mg/dL in men and women with arterial hypertension, respectively.

The calculator of the 10-year ASCVD risk is available for download as a supplemental Microsoft® Excel® file. This tool automatically calculates and graphically displays the ASCVD risk percentile for the corresponding sex and age (Central Figure). The user can also estimate the total cholesterol level when the patient is taking lipid-lowering drugs.

**Central Illustration:** : Determining Percentiles of Atherosclerotic Cardiovascular Risk According to Sex and Age in a Healthy Brazilian Population

Clinical application of the Microsoft Excel tool to calculate the 10-year risk of atherosclerotic cardiovascular disease and the percentile for sex and age. In the example depicted, the 10-year risk is not high, but the percentile for sex and age is greater than 75^th^. It is also possible to predict future 10-year risk if the patient persists at the same percentile. Percentiles may facilitate risk communication, increase patient awareness, help the physician advise the patient, and contribute to shared treatment decisions.

## Discussion

We established sex- and age-specific percentiles of the 10-year ASCVD risk distribution, according to the ACC/AHA pooled cohort equations, in a large sample of the Brazilian population. We found that most males up to 47 years and females up to 59 years above the 75^th^ risk percentile had a 10-year risk < 5% (low-risk category), a threshold frequently used to defer statin therapy in primary prevention with LDL-c below 190 mg/dL.^2^ We also provided an easy-to-use calculator of the ASCVD risk and the corresponding percentile for sex and age, which may facilitate the clinical use of this approach (Central Figure).

This study may be placed in a broader context of how to efficiently communicate the estimated cardiovascular risk, which is critical for patient engagement and successful implementation of preventive actions, especially in the scenario of shared decisions. Many people may see the absolute risk as an abstract, nonsense number. In addition, misperception of risk is common, including among individuals who attended our service.^12^ From the patient´s perspective, knowing how his/her risk compares to peers may increase risk awareness when the percentile is high.

Another proposal to facilitate the understanding of cardiovascular risk is to calculate the "heart age" or "vascular age" as synonymous with the age of someone of the same sex with the same predicted risk but all risk factors in normal ranges.^13,14^ Indeed, expressing the risk as the heart age has shown to be effective in controlling risk factors compared to informing the absolute risk or conventional medical advice.^15^ The vascular age, however, may be criticized for being a gross calculus, given that it is based on arbitrarily chosen normal values. Moreover, the concept of heart age does not involve a direct comparison with peers. Therefore, vascular age and risk percentiles are intrinsically different, and both are complementary in the task of optimizing risk communication.

In the context of cardiovascular prevention, risk percentiles share similarities with age-, sex-, and race/ethnicity-specific CAC score percentiles, which may guide clinical decisions.^16^ Similarly, the knowledge of the risk percentile may refine patient care, helping the healthcare provider decide on the intensity of preventive strategies, frequency of medical/laboratory evaluation, or the necessity of complementary risk assessment, e.g., investigation of subclinical atherosclerosis.

Risk percentiles may be particularly useful in young people whose calculated 10-year risk may be low even in the presence of risk factors, because age is the main determinant of risk. Our analyses identify men in their forties and women up to approximately 60 years as age groups in which a high risk percentile is more often associated with low 10-year risk. Proposals to guide preventive measures such as statin therapy in low 10-year risk individuals include the estimation of long-term risk and reducing the 10-year risk threshold to classify young persons as high-risk.^14,17^ The use of risk percentiles based on the 10-year risk distribution, as a way to indirectly mirror the lifetime risk, may be seen as another attractive possibility. Moreover, we showed that individuals in the low risk/high risk percentile frequently have excess weight and other metabolic abnormalities, consistent with the evidence linking metabolic syndrome and ASCVD risk.^18^ Conversely, these individuals often do not have very elevated LDL-c, especially in the presence of smoking or arterial hypertension. Therefore, a strategy of statin initiation solely based on very high LDL-c levels or 10-year risk thresholds would exclude many persons at high long-term risk. This issue becomes more relevant in the context of calls to preferentially use lifetime instead of 10-year risk prediction and treat ASCVD risk more intensively and earlier in life.^17,19^

Based on the pooled cohort equations, Navar et al. established ASCVD risk percentiles for sex, age, and race in the USA population.^4^ Some differences with our results can be noted. For example, Navar et al. reported the following 25^th^, 50^th^, and 75^th^ percentiles for 55-year-old non-Black males: 4.9%, 7.0%, and 10.2%, respectively. In our population, the respective percentiles were 4.7%, 6.0%, and 7.8%. In the USA population, the 25^th^, 50^th^, and 75^th^ percentiles for 65-year-old non-Black females were 5.1%, 6.9%, and 9.9%, respectively, while the respective numbers were 4.6%, 5.4%, and 7.4% in our population. Although the two studies used different methodological approaches to determine the percentiles, these differences may reflect a truly diverse risk distribution in the populations, reinforcing the limitations of extrapolating results from one country to another.

A relevant issue in our analysis refers to the method to deal with participants on lipid-lowering drug. Unlike antihypertensive medication, use of lipid-lowering drugs is not a variable in the pooled cohort equations, as this therapy was relatively uncommon in the derivation cohorts.^5^ Simply excluding those individuals from our analyses would act against the purpose of the study, that is, determining the risk percentiles in the whole target population. Imputing the total cholesterol value on medication into the risk equation could be misleading, underestimating the true risk. Thus, we opted to use an estimated total cholesterol level without medication in the risk calculation, as recommended by the most recent Brazilian guideline on dyslipidemia.^1^ We think that this approach was the most suitable for establishing population risk percentiles that may help clinicians decide whether statin therapy should be initiated, one of the main practical uses of ASCVD risk stratification.

Our study has the strength of evaluating a large, contemporary sample, which allowed us to conduct sex- and age-specific analyses. However, several limitations must be highlighted. First, the pooled cohort equations were neither validated nor calibrated in the Brazilian population. In particular, the independent effects of race, a key component of the pooled cohort equations, on cardiovascular events in Brazil are largely unknown. Lotufo and Benseñor reported higher age-adjusted death rates from total cardiovascular disease and stroke in Black persons;^20,21^ however, these analyses were not adjusted for other risk factors such as arterial hypertension, total cholesterol, and smoking. Second, our sample, which mostly comprised White individuals of high socioeconomic status who attended a private service in a large city in the Southeast Region, is far from being representative of the Brazilian population. Several studies report a lower burden of lipid abnormalities and high blood pressure in subgroups with higher socioeconomic or educational levels in Brazil,^22-27^ which may be at least partially explained by the more frequent use of preventive medications.^28^ In our study, this treatment effect is probably attenuated as antihypertensive medication is already a variable in the pooled cohort equations, and we applied a conversion factor to estimate total cholesterol level without medication. Smoking rates in Brazil are also lower as years of schooling increase.^26,27^ We also acknowledge that there are regional differences in the distribution of risk factors in the population.^22,26,27^ All these factors potentially modify the risk percentiles; therefore, caution must be exercised when applying the results of this study in other scenarios. Finally, cardiovascular risk estimation by scores is only the first step in risk stratification. Clinicians should bear in mind that several factors not contemplated in the risk equations, such as obesity, family history of premature ASCVD, inflammatory markers, and CAC may be used to identify at-risk persons.

## Conclusions

We established percentiles of the distribution of the 10-year ASCVD risk for sex and age in a large, although not representative sample of the Brazilian population. Most males up to 47 years and females up to 59 years above the 75^th^ percentile are categorized as low-risk individuals by the 2018 AHA/ACC Guideline on the Management of Blood Cholesterol. Persons in the low risk/high risk percentile frequently have metabolic abnormalities and not very elevated LDL-c levels. These individuals very likely have high long-term ASCVD risk and may be candidates for more aggressive risk factor control, including early initiation of statin therapy. Expression of risk percentiles may improve risk communication, increase patient awareness and adherence to preventive strategies, and facilitate shared decision-making processes.
